# Supplementary material for: Reconciling Mining with the Conservation of Cave Biodiversity: A Quantitative Baseline to Help Establish Conservation Priorities
Source: PLoS One. 2016 Dec 20;11(12):e0168348. doi: 10.1371/journal.pone.0168348 (PMC5173368; doi:10.1371/journal.pone.0168348)
Supplement: S1 Dataset — (ZIP) [file pone.0168348.s002.zip › Taxa/Serra Norte/SN_2013/N4E/Lista N4E-50.pdf]

CAVIDADE N4E-0050

Classe	Ordem	Fam/Outros	Gên/Outros	Espécie	S-U	U-S	Zon.
Annelida	Oligochaeta			jovem		1	E
Arachnida	Acari	Laelapidae		sp.69		X	E
Arachnida	Araneae	Araneidae		jovem		X	E
Arachnida	Araneae	Corinnidae		sp.2		1	E
Arachnida	Araneae	Ctenidae	*Enoploctenus*	sp.1		1	E
Arachnida	Araneae	Filistatidae		sp.1	X	X	E
Arachnida	Araneae	Ochyroceratidae		jovem		X	E
Arachnida	Araneae	Pholcidae	*Leptopholcus*	sp.1		X	E
Arachnida	Araneae	Pholcidae	*Mesabolivar*	sp.1	X	X	E
Arachnida	Araneae	Salticidae		sp.5	1		E
Arachnida	Araneae	Scytodidae	*Scytodes*	jovem	1		E
Arachnida	Araneae	Theridiosomatidae	*Plato*	sp.1		X	E
Arachnida	Pseudoscorpiones	Olpiidae		sp.1		X	E
Chilopoda	Geophilomorpha	Geophilidae		sp.1		1	E
Chilopoda	Scolopendromorpha	Scolopocryptopidae	*Tidops*	jovem		1	E
Diplopoda	Polyxenida	Polyxenidae		sp.1		X	E
Entognatha	Diplura	Campodeidae		sp.1		X	E
Insecta	Blattodea	Blaberidae		jovem	2		E
Insecta	Coleoptera			jovem		X	E
Insecta	Diptera	Aulacigastridae		sp.		X	E
Insecta	Diptera	Psychodidae	Phlebotominae	sp.		X	E
Insecta	Heteroptera	Reduviidae	Reduviinae	sp.	1	1	E
Insecta	Homoptera	Cixiidae		sp.3	X	X	E
Insecta	Hymenoptera	Formicidae	*Brachymyrmex*	sp.1	X		E
Insecta	Hymenoptera	Formicidae	*Camponotus*	sp.1		X	E
Insecta	Hymenoptera	Formicidae	*Camponotus*	sp.2	X		E
Insecta	Hymenoptera	Formicidae	*Gnamptogenys*	sp.1	X	X	E
Insecta	Hymenoptera	Formicidae	*Paratrechina*	sp.1		X	E
Insecta	Hymenoptera	Formicidae	*Solenopsis*	sp.1		X	E
Insecta	Isoptera	Rhinotermitidae	*Heterotermes*	sp.		X	E
Insecta	Isoptera	Termitidae	*Nasutitermes*	sp.		X	E
Insecta	Lepidoptera	Galacticidae		sp.		X	E
Insecta	Lepidoptera	Roeslerstammiidae		sp.		X	E
Insecta	Orthoptera	Phalangopsidae	*Paraclodes*	jovem	5	3	E
Malacostraca	Isopoda	Dubioniscidae		sp.1		X	E
Amphibia	Anura	Strabomantidae	*Pristimantis*	cf. *fenestratus*	2	5	E
Mammalia	Chiroptera	Emballonuridae	*Peropteryx*	*kappleri*	2	1	E
